# Outcomes of the electromagnetic navigation bronchoscopy using forceps for lung lesion suspected malignancy: A retrospective study

**DOI:** 10.1097/MD.0000000000035362

**Published:** 2023-10-20

**Authors:** Tae Hun Kim, Mi-Ae Kim, Hyun Jung Kim, Yong Shik Kwon, Jae Seok Park, Sun Hyo Park

**Affiliations:** a Division of Pulmonary and Critical Care Medicine, Department of Internal Medicine, Keimyung University Dongsan Medical Center, Daegu, South Korea.

**Keywords:** bronchus sign, cytology, diagnostic yield, electromagnetic navigation bronchoscopy, gross size of specimen, lung cancer

## Abstract

Many studies have reported electromagnetic navigation bronchoscopy (ENB) diagnostic yields and the importance of size and computed tomography (CT) bronchus sign. This study aimed to determine the diagnostic yield of ENB alone, using forceps biopsy and cytology. We analyzed the factors associated with yield and complications according to gross specimen size. This retrospective study included patients who underwent ENB using forceps for suspected lung lesions on CT between January 2020 and December 2022 in South Korea. Factors related to the ENB diagnostic yield and complications were evaluated, and the impacts of gross specimen size and cytology were analyzed. A total of 276 patients were analyzed. The final diagnostic yield was 75.5% after excluding indeterminate cases. Sensitivity and specificity were 74.2% and 100%, respectively. Pneumothorax developed in 1.4% (4/276) of cases, with no grade 3 or higher bleeding. Univariable analysis showed that the number of biopsies and the size of the gross specimen were related to the diagnosis. Multivariable analyses showed that a larger lesion size on CT was a significant factor for diagnosis. The gross size of the specimens was not significantly associated with epinephrine use. ENB had acceptable diagnostic yield and safety for diagnosing lung lesions with suspected malignancy. Obtaining more tissue through biopsy may not increase bleeding or pneumothorax complications. Identifying patients with lesion characteristics, including CT bronchus sign, would help increase ENB diagnostic yield.

## 1. Introduction

Lung cancer currently accounts for a large proportion of all cancer diagnoses (11.6% of all cancer diagnoses), and the mortality rate is also high (18.4% of all cancer-related mortalities).^[1]^ The recent main strategy for reducing mortality is early screening with low-dose computed tomography (CT).^[2]^ However, CT-based surgery was also shown in 18% to 34% of benign nodules. Therefore, pathological confirmation before surgery is important to reduce over-treatment.^[3]^ Recent guidelines for non-small-cell lung cancer have emphasized the importance of molecular testing for EFGR, ALK, and PD-L1.^[4]^ In addition, study on molecular testing, including next-generation sequencing technology, have been conducted,^[5]^ so the demand for tissue acquisition of peripheral lung lesions and initial diagnosis of early lung cancer is expected to increase further.

Biopsy methods for the peripheral pulmonary lesions have been developed, including computed tomography-guided lung biopsy and bronchoscopic biopsy. The sensitivity of CT-guided lung biopsy is over 85% in lesions over 2 cm,^[6]^ and the false-negative rate is under 10%.^[6]^ However, procedure-related complications, especially pneumothorax and hemorrhage, have been reported in up to 61% and 5% to 16.9%, respectively.^[6]^ And recently, peripheral pulmonary lesions biopsy methods using bronchoscopy, including radial endobronchial ultrasound, guide sheath, and electromagnetic navigation bronchoscopy (ENB), were developed.^[7]^ A previous meta-analysis reported a positive and definite diagnosis of approximately 65% and overall diagnostic accuracy of 74% and 38.5% to 96.8% for SuperDimension, and 33.0% to 90.2% for Veran.^[8,9]^ A prospective multicenter NAVIGATE reported 1-year diagnostic yields of 73% and confirmed malignancy in 44%.^[10]^ A recent study reported that not only the size of the target but also the present of computed tomography bronchus sign (CT-BS) is more likely to be diagnosed with guided bronchoscopy.^[11,12]^

To date, few studies have been performed with Veran and the analysis focused only on the diagnosis. No study has evaluated the number of biopsies, the amount of tissue, so there is no consensus on the optimal number of biopsies yet. This study performed ENB with forceps via Veran, and we aimed to show the factors associated with diagnostic yields, and the impact of the number of biopsies and gross specimen size on diagnostic yields and complications.

## 2. Materials and methods

### 2.1. Study design and patients

**Figure 1.**
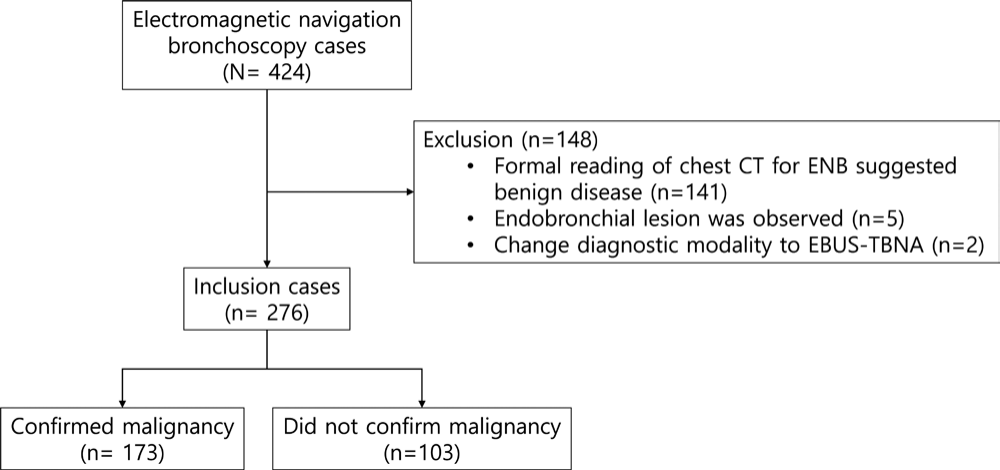
Flow diagram of the study population. CT = computed tomography, EBUS-TBNA, endobronchial ultrasound-guided transbronchial needle aspiration, ENB = electromagnetic navigation bronchoscopy.

This retrospective study analyzed the clinical data of 422 patients who underwent ENB at a tertiary medical center in South Korea between January 2020 and December 2022. We excluded 141 patients who were considered benign based on the final interpretation of the CT scan performed for ENB. In many cases, ENB was planned because of the abnormality of the CT performed at a local medical center. If the target size was reduced from the previous CT, it was considered benign, and all the cases were excluded. In addition, 5 patients with endobronchial lesions were excluded (Fig. 1).

### 2.2. ENB procedure

For the ENB procedure, all patients planned for ENB with inspiration and expiration chest CT to mark the biopsy target and reconstruct airway routes before the procedures via the navigation platform. All ENB procedures were conducted with moderate sedation by intravenous 2 to 5 mg of midazolam and 25 to 50 ug of fentanyl at the procedure onset. Additional doses of midazolam or fentanyl were administered when adequate sedation was not achieved.

All ENB procedures were performed by 1 of 6 pulmonologists, each with at least 2 years of experience in bronchoscopy (T.H.K., M.A.K., H.J.K., Y.S.K., J.S.P., and S.H.P.). The Spin Thoracic Navigation System (SYS-4230 K; Veran Medical, St. Louis, MO) with bronchoscope outer diameters of 6.0 mm (BF-1TQ290), 4.9 mm (BF-260), and 4.2 mm (BF-P290) (Olympus Corporation, Tokyo, Japan) were used in all procedures. All ENB cases used forceps only for biopsy without an aspiration needle.

### 2.3. Data collection

Data were collected and analyzed retrospectively. Information was obtained from electronic medical records. The following data were recorded and analyzed: demographic characteristics including sex, age, height and weight, body mass index, comorbidities, and smoking history, results of lung function test; forced expiratory volume in 1 second, forced vital capacities, and forced expiratory volume in 1 second/forced vital capacities, radiologic characteristics of chest CT reports from the radiologist including lesion size on CT, types of lesions such as solid and sub-solid, whether pleural abutting, lobar locations, and axial distribution, number of biopsies, usage of epinephrine for hemostasis, adverse events including pneumothorax, pathologic reports including diagnosis, and gross specimen size, and report of cell block of washing specimen.

Regarding the test learning curve, the first (January 2020–December 2021 [n = 125]) and second (January 2022–December 2022 [n = 151]) halves were compared according to time. The presence of CT-BS has been classified in previous studies.^[11,13]^ In this study, the presence of CT-BS (airway sites adjacent to the target, directly aligned to the target) or not (absent) was classified. The axial distribution was classified into the inner 1/3, middle, and outer 1/3.^[14]^ Vertical distribution was classified into upper (right and left upper lungs) and lower (right middle lung, right lower lung, and left lower lung) lungs. The lesion size on CT was measured as the longest diameter. The lesion type was classified as solid, sub-solid, or ground-glass opacity; however, no participants had ground-glass opacities. The number of biopsies was defined as the number of biopsies from which tissue was obtained. The gross specimen size was recorded by collecting the obtained tissue samples and measuring the approximate size by a lung cancer-specialized pathologist as A cm × B cm × C cm and analyzed as A × B × C cm^3^ at the time of analysis.

### 2.4. Statistical analysis

Data were presented as frequencies (%) for categorical variables and as mean (± standard deviation) for continuous variables. The diagnostic yield was calculated for each case as the sum of true positives and true negatives (TN) rates.^[10]^ Among the unconfirmed cases, TNs were defined as improved lung lesions after a follow-up of at least 6 months, confirmed tuberculosis, improvement after anti-tuberculosis medication, or surgically proven granuloma. Cases with initial negative results and insufficient information due to follow-up loss or refusal to undergo further workup were classified as indeterminate. It was included in the sensitivity analysis, assuming all were TN or false negatives. Low and high estimates of the diagnostic yield, sensitivity, specificity, positive predictive value, and negative predictive value were obtained.

A chi-square test was performed to evaluate the association between 2 nominal variables. A linear-by-linear test was used to assess the association between variables and ordered categories. The Student *t* test was used for descriptive analysis. The Mann–Whitney *U* test was used to test the association between variables that did not follow a normal distribution. Pearson correlation coefficient was used to analyze the association between 2 continuous variables. Logistic regression analysis was used to evaluate pathologic confirmation factors. Variables with a *P* value < .2 in the univariable analysis were entered into the multivariable analysis and selected by the backward log-likelihood ratio method. Since the number of biopsies was determined by the operator, we did not enter a multivariable analysis. A dot plot was used to visualize the relationship between 2 continuous variables. A *P* value of < .05 was considered statistically significant. All statistical analyses were performed using SPSS Statistics version 25 (IBM Corp., Armonk, NY) and R version 4.1.1.

### 2.5. Statement of ethics

The study protocol was reviewed and approved by the Institutional Review Board of the Keimyung University College of Medicine (approval no. 2023-03-055). This study conformed to the principles of the Declaration of Helsinki (revised edition 2013). The requirement for informed consent was waived due to the retrospective nature of this study.

## 3. Results

### 3.1. Participant and baseline characteristics

Figure 1 shows the results of 276 ENB cases that were evaluated. Table 1 summarizes the characteristics of the participants. The average age was 72.0 (±9.9) year-old; 87 (31.5%) were ever-smokers, and 195 (70.7%) patients were male. The mean lesion size on CT was 3.3 cm (±1.8), and 53 (19.2%) were subsolid nodules. CT-BS was showed on 226 (81.9%) patients (Table 1).

**Table 1:** Baseline characteristics of the patients.

Characteristics	Total (N = 276)	Not confirmed Malignancy (n = 103)	Confirmed malignancy (n = 173)	*P* value
Age	72.0 (±9.9)	70.3 (±9.2)	73.1 (±10.1)	.023
Male sex	195 (70.7%)	65 (63.2%)	130 (75.1%)	.034
BMI	23.8 (±3.9)	23.3 (±3.5)	23.8 (±4.2)	.883
Ever smoking	87 (31.5%)	28 (27.2%)	59 (34.1%)	.231
Pulmonary function test				
FEV1/FVC	0.71 (±0.10)	0.73 (±0.10)	0.70 (±0.10)	.046
FEV1 (% of predicted)	92.6 (±22.1)	93.6 (±20.5)	92.0 (±23.0)	.574
FVC (% of predicted)	88.3 (±15.7)	88.3 (±15.5)	88.4 (±15.9)	.979
Comorbidity				
Hypertension	138 (50.0%)	47 (45.6%)	91 (52.6%)	.263
Diabetes mellitus	64 (23.2%)	21 (20.4%)	43 (24.9%)	.395
Airway disease	71 (25.7%)	20 (19.4%)	51 (29.5%)	.064
Interstitial lung disease	19 (6.9%)	6 (5.8%)	13 (7.5%)	.592
Lobar location				
Right upper lung	90 (32.6%)	29 (28.2%)	61 (35.3%)	.223
Right middle lung	24 (8.7%)	8 (7.8%)	16 (9.2%)	.673
Right lower lung	60 (21.7%)	28 (27.2%)	32 (18.5%)	.091
Left upper lung	65 (23.6%)	22 (21.4%)	43 (24.9%)	.508
Left lower lung	36 (13.0%)	14 (13.6%)	22 (12.7%)	.835
Axial distribution*				.423
Inner	18 (6.5%)	5 (4.9%)	13 (7.5%)	
Middle	102 (37.0%)	35 (34.0%)	67 (38.7%)	
Outer	156 (56.5%)	63 (61.2%)	93 (53.8%)	
Lesion size on CT, cm	3.3 (±1.8)	2.8 (±1.2)	3.6 (±2.0)	<.001
Type of lesion, n (%)				
Solid	223 (80.8%)	79 (76.7%)	144 (83.2%)	.182
Sub-solid	53 (19.2%)	24 (23.3%)	29 (16.8%)	
Pleural abutting	72 (26.1%)	26 (25.2%)	46 (26.6%)	.805
Cavity	32 (11.6%)	10 (9.7%)	22 (12.7%)	.450
CT-BS	226 (81.9%)	66 (64.1%)	160 (92.5%)	<.001
Biopsy specimen				
Numbers of biopsies, n	11.2 (±5.8)	8.8 (±5.4)	12.4 (±5.6)	<.001
Gross size of specimen†, cm^3^	0.11 (±0.09)	0.08 (±0.06)	0.12 (±0.09)	.001
Bleeding control				
Epinephrine, n	2.9 (1.9)	2.9 (1.9)	2.9 (2.0)	.919

Data are expressed as mean (±standard deviation) or n (%).

BMI = body mass index, CT-BS = computed tomography-bronchus sign, FEV1 = forced expiratory volume in 1 second, FVC = forced vital capacities.

* Axial distribution was defined as inner 1/3, middle 1/3, and outer 1/3.

† Gross size of the specimen was measured approximately by a pathologist as A cm × B cm × C cm and calculated as A × B × C cm^3^.

Between the confirmed and unconfirmed cases, the confirmed group was significantly older, had more males, a larger lesion size on CT, more present CT-BS, and a greater number of biopsies with larger specimen sizes. The lobar and axial distribution, type of lesion, pleural abutting, and cavity were not significantly different (Table 1).

### 3.2. Diagnostic outcomes and complications

**Figure 2.**
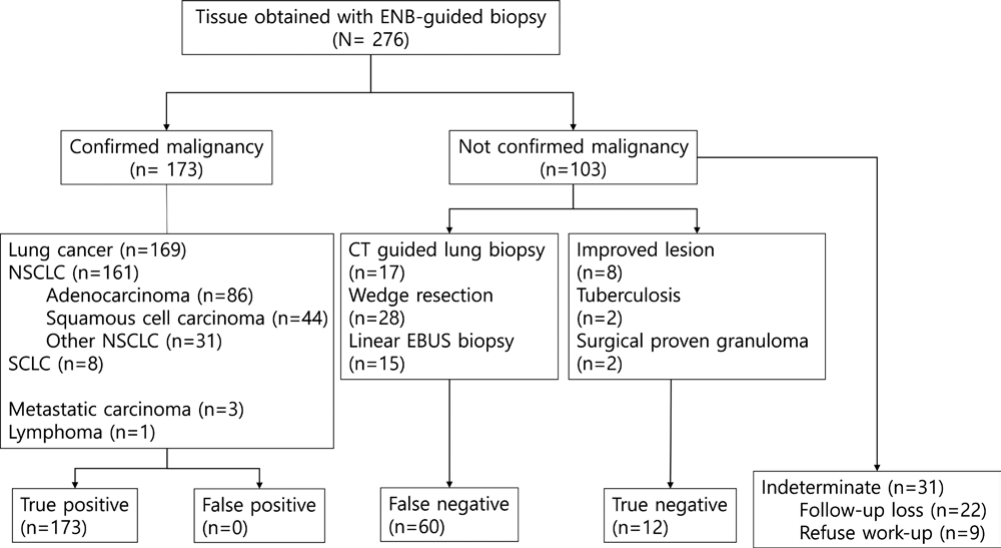
Diagnostic results of participants. CT = computed tomography, EBUS = endobronchial ultrasonography, ENB = electromagnetic navigation bronchoscopy, NSCLC = non-small cell lung cancer, SCLC = small cell lung cancer.

The final diagnostic results, including those not confirmed in ENB cases, are shown in Figure 2. Malignancy was confirmed via ENB in 173 of 276 (62.7%) patients. Of the patients without confirmed malignancy, 60 were diagnosed with lung cancer using other methods including CT-guided percutaneous lung biopsy. Twelve cases were true-negative (Fig. 2). The final diagnostic yield of ENB was 75.5%, and the sensitivity and specificity were 74.2% and 100.0%, respectively (Table 2).

**Table 2:** Diagnostic yield of electromagnetic navigation bronchoscopy.

	Excluding indeterminate cases (n = 245)	Low estimate (n = 276)	High estimate (n = 276)
Diagnostic yield [TP + TN/All cases]	185/245 (75.5%)	185/276 (67.0%)	216/276 (78.2%)
Sensitivity for malignancy [TP/TP + FN]	173/233 (74.2%)	173/264 (65.5%)	173/233 (74.2%)
Specificity for malignancy [TN/TN + FP]	12/12 (100.0%)	12/12 (100.0%)	43/43 (100.0%)
Positive predictive value [TP/TP + FP]	173/173 (100.0%)	173/173 (100.0%)	173/173 (100%)
Negative predictive value [TN/TN + FN]	12/72 (16.7%)	12/103 (11.6%)	43/103 (41.7%)

Data are expressed as n (%).

FN = false negative, FP = false positive, TN = true negative, TP = true positive.

Pneumothorax developed in 1.4% (4/276) of the patients, and chest tube insertion was required in all patients. The number of biopsies and gross specimen size showed no statistically significant relationship with pneumothorax (Supplemental digital content [Table S1]), http://links.lww.com/MD/K67. There was no grade 3 or higher bleeding after the biopsy, according to the proposed grade.^[15]^ None of the patients required intensive care or died of procedure-related complications. There was no significant relationship between the gross size of the specimen and the use of epinephrine for hemostasis (Pearson correlation, −0.112; *P* = .177) (Supplemental digital content [Table S2], http://links.lww.com/MD/K68, Supplemental digital content [Fig. S1]), http://links.lww.com/MD/K69.

### 3.3. Factors affecting higher diagnostic yield and the value of cytology

Univariable and multivariable analyses of the factors associated with the diagnosis are shown in Table 3. In the univariable analysis, older age, male sex, larger lesion size on CT, presence of CT-BS, many biopsies performed (odds ratio [OR] 1.147; *P* = .001), and larger gross specimen size (OR 909.184; *P* = .001) were associated with a higher diagnostic yield. When analyzing the relationship between the number of biopsies and the gross size of the specimen, a positive correlation was observed (Pearson correlation 0.546; *P* < .001) (Supplemental digital content [Table S2]), http://links.lww.com/MD/K68. In multivariable analysis, only a larger lesion size on CT (OR 1.260; *P* = .019) and CT-BS (OR 6.042, *P* < .001) were significant factors for diagnosis (Table 3).

**Table 3:** Logistic regression model for pathologic confirmation.

Characteristic	Univariable analysis			Multivariable analysis		
	OR	95% CI	*P* value	OR	95% CI	*P* value
Age	1.029	1.004–1.056	.024	1.025	0.997–1.054	.086
Male sex	1.767	1.042–2.998	.035	1.519	0.848–2.723	.160
Ever smoking	0.937	0.265–3.304	.919			
BMI (kg/m^2^)	1.005	0.940–1.075	.883			
Type of lesion (sub-solid)	0.663	0.361–1.216	.184	0.547	0.280–1.068	.077
Cavity	1.355	0.614–2.988	.452			
Lesion size on CT, cm	1.405	1.167–1.691	<.001	1.260	1.038–1.528	.019
CT-BS	6.900	3.447–13.340	<.001	6.042	2.911–12.541	<.001
Vertical distribution (lower)	0.720	0.441–1.178	.191	0.744	0.429**–**1.293	.294
Axial distribution (outer)	0.568	0.193–1.672	.304			
Biopsy numbers, n	1.147	1.060–1.242	.001			
Gross size of specimen, cm^3^	909.184	16.569–49,889.056	.001			

BMI = body mass index, CI = confidence interval, CT-BS = computed tomography-bronchus sign, OR = odds ratio.

There was no significant difference in the learning curve between early and recent cases (*P* = .871). The numbers of confirmed malignancies were 79 (63.2%) and 94 (62.5%), respectively (Supplemental digital content [Table S3]), http://links.lww.com/MD/K70. Among the cytological results (n = 261), even among the cases pathologically confirmed via ENB, only 50.0% presented with atypical or malignant cells, suggesting a malignant tumor (Supplemental digital content [Table S4]), http://links.lww.com/MD/K71.

## 4. Discussion

This study retrospectively evaluated the diagnostic yield of ENB using forceps alone. The diagnostic yield of ENB was 75.5%. Therefore, ENB may be an effective diagnostic tool for lung lesions. The rate of pneumothorax complications was low (1.4%), similar to a previous study that reported 2.0% to 2.1%^[12,16]^ to 10%.^[17]^ Multivariable analysis revealed that a larger lesion size on CT and the presence of CT-BS were significant factors associated with pathological confirmation. Among the CT-BS cases, pathological confirmation was obtained in 70.9% (160/226) cases. Most studies report diagnostic yields between 67% and 84%.^[8,18,19]^ The NAVIGATE multicenter study on ENB reported an overall diagnostic yield of 72.9%.^[10]^ To date, limited data is available on the Spin Thoracic Navigation System. A few studies on ENB usefulness and safety have reported 33.0% to 90.2% for Veran.^[9,12]^ This study provided valuable reports of real world data on the diagnostic yields and safety of 276 extensive cases using the Spin Thoracic Navigation System.

Based on cytology, only 42.6% (92/216) of the patients reported atypical or malignant cells among those finally confirmed with malignancy. And malignant cells were observed in 19.4% (42/216) of patients. Among the cases of malignancy confirmed by ENB, only 50% were reported as atypical or malignant cells, which is higher than that reported in a previous study with a sensitivity of 20%.^[20]^ Although bronchoalveolar lavage was obtained from the target lesion using ENB, this study showed the limited value of cytology tests and obtaining cell block specimen even through ENB.

Previous ENB studies have identified various factors, such as size, location, CT-BS, additional use of radial endobronchial ultrasound,^[21]^ and user experience, but the results varied among the studies.^[10,22–25]^ Consistent with previous studies, our study also showed that a larger lesion size on CT and the presence of CT-BS were significant factors for diagnostic yields. And there was a tendency for more pathologic confirmed via ENB to occur on the side where more biopsies were performed and there was no significant relationship between obtaining more gross specimens and complications. Therefore, this study suggests that ENB biopsy may be useful for obtaining more tissue. Furthermore, it might be judged that the learning curve of ENB was not as long as that of surgery if ENB was performed by an experienced bronchoscopist. Regarding respiratory variation, contrary to expectations, the diagnostic rate was not significantly lower in the lower lobe, and the diagnostic rate of lesions in the outer 1/3 was not lower than that in the inner 2/3. The complication risk of pneumothorax was 1.4%, as low as in previous studies,^[10,26]^ and markedly lower compared with reports of CT-guided lung biopsy, with a risk of 19% to 25%.^[27,28]^

Our study has several limitations. First, this was a retrospective study conducted at a single center. Therefore, the results require further validation, and a more extensive study is required. Second, in our study, 6 bronchoscopists performed ENB, so the propensity during the procedure including the biopsy number could be slightly different; however, this was not analyzed separately. There were limitations in evaluating the diagnostic yields according to the biopsy specimen. Third, our data could only include ENB using forceps. Therefore, the value of needle aspiration biopsy was not analyzed. Despite these limitations, our study attempted to analyze as many participants as possible with various factors that might be related to yields. This study analyzed factors, including the number of biopsies and cytology, which have not yet been studied sufficiently. Therefore, this study provides a meaningful report on forceps biopsy using ENB.

In conclusion, ENB had an acceptable diagnostic yield and safety for diagnosing lung lesions suspected to be malignant. Obtaining more tissue through biopsy may not increase complications. To increase the ENB diagnostic yield, it would be helpful to identify patients with lesion characteristics, including CT-BS.

## Author contributions

**Conceptualization:** Tae Hun Kim, Yong Shik Kwon, Jae Seok Park, Sun Hyo Park.

**Data curation:** Tae Hun Kim.

**Formal analysis:** Tae Hun Kim, Hyun Jung Kim, Sun Hyo Park.

**Methodology:** Tae Hun Kim, Mi-Ae Kim.

**Writing – original draft:** Tae Hun Kim, Jae Seok Park.

**Writing – review & editing:** Tae Hun Kim, Mi-ae Kim, Hyun Jung Kim, Yong Shik Kwon, Jae Seok Park, Sun Hyo Park.

## Supplementary Material










