# Supplementary figures and images for: Outcomes of the electromagnetic navigation bronchoscopy using forceps for lung lesion suspected malignancy: A retrospective study
Source: Medicine (Baltimore). 2023 Oct 20;102(42):e35362. doi: 10.1097/MD.0000000000035362 (PMC10589535; doi:10.1097/MD.0000000000035362)

**Supplemental digital content**

**Fig. S1.** Dot plot of gross size of specimen and use of epinephrine


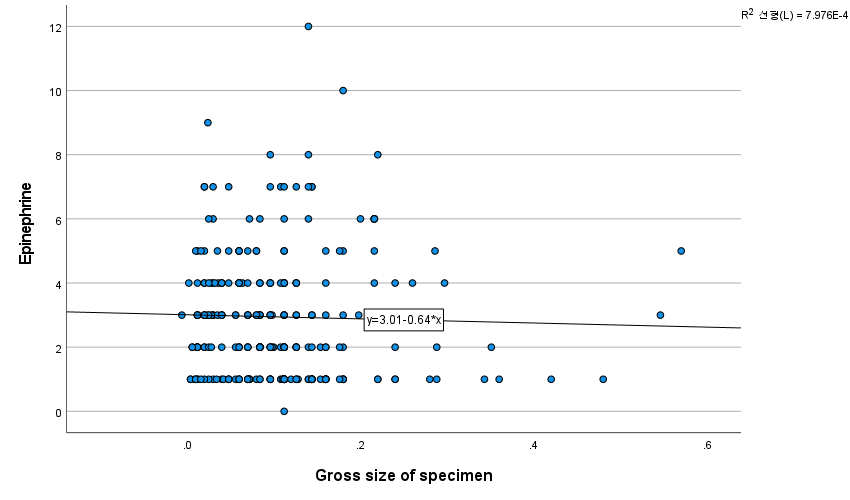

Supplement: Supplementary file 1 [file medi-102-e35362-s001.docx]
