# Supplementary material for: Outcomes of the electromagnetic navigation bronchoscopy using forceps for lung lesion suspected malignancy: A retrospective study
Source: Medicine (Baltimore). 2023 Oct 20;102(42):e35362. doi: 10.1097/MD.0000000000035362 (PMC10589535; doi:10.1097/MD.0000000000035362)
Supplement: Supplementary file 2 [file medi-102-e35362-s002.docx]

**Supplemental digital content**

**Table S2.** Correlation between the number of biopsies, gross size of the specimen, and use of epinephrine

| Number of biopsies |  | Number of biopsies | Gross size of specimen | Use of epinephrine, n |
| --- | --- | --- | --- | --- |
|  | Pearson correlation | 1 | 0.546 | -0.112 |
|  | Significance (2-tailed) |  | <0.001 | 0.177 |
| Gross size of specimen |  | Number of biopsies | Gross size of specimen | Use of epinephrine, n |
|  | Pearson correlation | 0.546 | 1 | -0.028 |
|  | Significance (2-tailed) | <0.001 |  | 0.648 |
