# Supplementary material for: Outcomes of the electromagnetic navigation bronchoscopy using forceps for lung lesion suspected malignancy: A retrospective study
Source: Medicine (Baltimore). 2023 Oct 20;102(42):e35362. doi: 10.1097/MD.0000000000035362 (PMC10589535; doi:10.1097/MD.0000000000035362)
Supplement: Supplementary file 4 [file medi-102-e35362-s004.docx]

**Supplemental digital content**

**Table S3.** Relation between examination date and diagnostic yield

|  | Not confirmed  (n=103) | Confirmed  (n=173) |
| --- | --- | --- |
| January 2020 to December 2021 (n=125) | 46 (36.8%) | 79 (63.2%) |
| January 2022 to December 2022 (n=151) | 57 (37.7%) | 94 (62.5%) |

0 cells have been expected count less than 5.

Pearson Chi-square significance was *P*=0.871
