# Supplementary material for: Outcomes of the electromagnetic navigation bronchoscopy using forceps for lung lesion suspected malignancy: A retrospective study
Source: Medicine (Baltimore). 2023 Oct 20;102(42):e35362. doi: 10.1097/MD.0000000000035362 (PMC10589535; doi:10.1097/MD.0000000000035362)
Supplement: Supplementary file 5 [file medi-102-e35362-s005.docx]

**Supplemental digital content**

**Table S4.** Pathologic diagnosis and cytology results

|  | Not confirmed  (n=93) | Confirmed  (n=168) |
| --- | --- | --- |
| Negative for malignancy | 84 (90.3%) | 84 (50.0%) |
| Atypical cell | 8 (8.6%) | 43 (25.6%) |
| Malignant cell | 1 (1.1%) | 41 (24.4%) |

0 cells have been expected count less than 5.

Linear-by-linear test was done, Pearson Chi-square significance was *P*<0.001
